# Additive Manufacturing of Head Surrogates for Evaluation of Protection in Sports

**DOI:** 10.3390/polym16121753

**Published:** 2024-06-20

**Authors:** Ramiro Mantecón, Borja Valverde-Marcos, Ignacio Rubio, George Youssef, José Antonio Loya, José Díaz-Álvarez, María Henar Miguélez

**Affiliations:** 1 Department of Mechanical Engineering, Universidad Carlos III de Madrid, Avenida de la Universidad, 30, Leganés, 28911 Madrid, Spain; bvalverd@ing.uc3m.es (B.V.-M.); igrubiod@pa.uc3m.es (I.R.); jodiaz@ing.uc3m.es (J.D.-Á.); mhmiguel@ing.uc3m.es (M.H.M.); 2 Experimental Mechanics Laboratory, San Diego State University, 5500 Campanile Drive, San Diego, CA 92182, USA; gyoussef@sdsu.edu; 3 Department of Continuum Mechanics and Structural Analysis, Universidad Carlos III de Madrid, Avenida de la Universidad, 30, Leganés, 28911 Madrid, Spain; jloya@ing.uc3m.es

**Keywords:** head surrogates, head impacts, biomechanics, helmet testing, additive manufacturing

## Abstract

Head impacts are a major concern in contact sports and sports with high-speed mobility due to the prevalence of head trauma events and their dire consequences. Surrogates of human heads are required in laboratory testing to safely explore the efficacy of impact-mitigating mechanisms. This work proposes using polymer additive manufacturing technologies to obtain a substitute for the human skull to be filled with a silicone-based brain surrogate. This assembly was instrumentalized with an Inertial Measurement Unit. Its performance was compared to a standard Hybrid III head form in validation tests using commercial headgear. The tests involved impact velocities in a range centered around 5 m/s. The results show a reasonable homology between the head substitutes, with a disparity in the impact response within 20% between the proposed surrogate and the standard head form. The head surrogate herein developed can be easily adapted to other morphologies and will significantly decrease the cost of the laboratory testing of head protection equipment, all while ensuring the safety of the testing process.

## 1. Introduction

Head trauma events resulting from blunt-force impacts comprise impulsive loads that result in rapid accelerations of the head and jolting of the brain matter. The repercussions of head impacts may be categorized into skull fractures or different typologies of internal injuries (e.g., focal injuries with regions of affection and diffuse injuries with lesions extended globally to the brain) [1]. The latter encompass diffuse axonal injuries and traumatic brain injuries (TBIs), commonly referred to as concussions, which are highly prevalent in sports due to the risks of head impacts [2]. Sports activities such as contact sports (football, rugby, hockey, etc.) and individual sports with high-speed mobility (e.g., cycling or skiing) are prone to head impacts at different energies, orientations, and frequencies [3]. To mitigate the risks associated with head impacts, the governing organizations of these sports and healthcare practitioners mandate protective head equipment. However, developing optimal impact mitigation mechanisms is impeded by three interrelated factors: (1) the in vivo testing of head impacts is unfeasible and unethical in severe impact scenarios, (2) the ex vivo behavior of biological tissues differs from their response in a real-world environment irrespective of researchers’ efforts to maintain the freshness of the tissues, and (3) biological tissues exhibit a high degree of variability in their mechanical response due to age, gender, and ethnicity [4]. Hence, the primary motivation of this research is to develop an in vitro head surrogate that transcends the above-listed limitations while allowing for further exploration of head impacts and protective equipment. Such efforts are imperative for quickly translating novel protective equipment from the laboratory to the field.

The pursuit of substitutes for human or animal models, including cadavers and animal tissues, has led to the design and construction of head surrogates with a broad range of fidelities and complexities. According to Kneubuehl’s definition concerning ballistic scenarios, a surrogate is a construct with comparable geometrical and mechanical qualities that should react to an impact in a similar way to human tissue in terms of elasticity and fracture energy [5], allowing the exploration of the physics and mechanics of the impact to be readily and ethically carried out. Surrogates should also accurately replicate the biomechanical behavior of human tissue at different lengths and time scales. For example, studies of concussion biomechanics are conducted on anthropomorphic testing devices (ATDs), while impact tests have been performed on dummies for decades in the automotive field. The Hybrid III head form (Humanetics) is ubiquitous in laboratory testing since it exhibits a biomechanical response representative of an adult male in the 50th percentile, and is utilized in the automotive industry, military safety, and sports biomechanics. The development of the Hybrid III head and neck dummy was initially proposed by Hubbard and McLeod for vehicular impact testing [6]. Although its dimensions and mass are faithful to the 50th percentile of adult males, it has undergone simplification in terms of geometric attributes. The original instrumentation of the model comprises a set of accelerometers to enable the calculation of the HIC (Head Injury Criterion) [7]. The HIC value was employed to ascertain the crashworthiness of vehicles and the safety of passengers during the development of new cars [8]. Subsequently, the Hybrid III ATD was employed in a broader range of biomechanical testing, including the effects of gripping devices and airbag interactions [9].

In the early 2000s, Pellman and Viano conducted numerous Hybrid III American football impact reconstruction trials to infer the loading conditions leading to concussion from helmet-to-helmet and helmet-to-ground impacts [10]. Pellman and co-investigators clinically and biomechanically assessed concussions (mild TBI) from the available American football data [11]. Pellman et al. vigorously studied recordings of in-game situations that resulted in player concussions, inferring the kinematics of collision scenarios and establishing the threshold values of concussion probability [12,13]. Pellman’s criterion was subsequently used to study the implications of different impact scenarios in laboratory settings using instrumented dummies [14].

Similarly, numerous studies have employed surrogates specifically designed to enhance the representativeness of surrogate elements. Zhang et al. investigated the biomechanical responses of the head under impact loads in the parietal–temporal region, utilizing skulls isolated from post-mortem human subjects (PMHS) and replacing the intracranial material with a surrogate brain based on Sylgard gel [15], where the gel and the skull were in direct contact. They computed the translational accelerations at the center of masses by taking accelerations in positions with a known distance to the center of masses and compensating the rotational accelerations. They reported good agreement between the different accelerometers at low velocities (close to 2.5 m/s). However, more considerable disparities were found in velocities close to 7 m/s, attributed to increased lateral skull deformation. This deformation induced up to 100% differences in the computed translational accelerations between different accelerometers (from 121 g to 224 g). Merkle et al. designed a human surrogate head model (HSHM) composed of biosimulant materials that replicated the skin, face, skull, and brain using 3D-printed molds [16], which was mounted on the neck of a Hybrid III model and instrumented with various sensors. The skull was constructed from a glass–epoxy mixture, the brain from Sylgard gel, and the facial structure from syntactic foam, and the assembly was subjected to blast loading using a shock tube. Their results evidenced response differences in the anterior and posterior regions of the head model. Additionally, while the magnitudes of the peaks depended on the loading conditions, the waveform characteristics of the pressure evolution were primarily determined by anatomical location. They also reported that using a helmet as protection mainly affected the negative phase of the pressure waveform, showing a smaller effect on the positive peaks. Hence, head surrogates with various fidelities represent a viable testbed for laboratory testing, especially when modified with different simulant materials and sensors. The research leading to this article builds on the ability of 3D printing to further explore the utility of head surrogates in biomechanical testing.

In a recent study, Banton et al. proposed a model with more reliable geometric definitions for studying shock wave reverberations in the head [17]. They used polymethyl methacrylate (PMMA) to simulate the skull, filled with a biogel, as a brain mass surrogate, and employed the simulator to calculate the intracranial pressure and stresses in the brain substitute in response to a primary impact. With this study, Banton et al. elucidated the importance of close emulation of the geometric attributes in correctly replicating the wave propagation and detecting its consequences. Another recent study at the University of Michigan employed a surrogate to study the cavitation phenomenon in brain sulci upon impact in a drop tower and a magnetic resonance scanner [18]. The proposed brain surrogate was based on two gelatins of different polyacrylamide concentrations to distinguish between gray and white matter, while the cranial bone material was represented by polylactic acid (PLA). The PLA/polyacrylamide surrogates enabled the measurement of the resulting pressures in the contrecoup region and the correlation with the cerebrospinal fluid cavitation (simulated by water).

Various safety standards and norms establish requirements for protective structures used in impact scenarios at different speeds and orientations. UNE-EN 1078 includes a set of mandatory standards for the design of helmets for cyclists and roller skates in Europe, while ASTM F1447 is its American analog [19,20]. Additionally, numerous research studies have assessed head accelerations and the absorption capacity of helmets in various impact scenarios. Cripton et al. studied the effect of helmets on impact efficacy under different loading scenarios, measuring acceleration and calculating peak acceleration and the Head Injury Criterion (HIC) to forecast the probabilities of brain injury [21]. The usefulness of protective equipment was demonstrated. In their work, Cripton et al. used a guided drop tower to impact the frontal region of the head using the 50th percentile male Hybrid III. They reported a broadening impact pulse of the helmeted cases and a significant reduction in the magnitude of the peak acceleration. Rush et al. tested American football helmets with facial-region protection elements using a guided drop tower to target specific impact orientations [22], reporting that the standard certification thresholds were not conservative enough given the variability of the testing setup and suggesting lowering the Severity Index threshold. Moreover, Bland et al. introduced a method for testing cycling helmets using a free-fall tower equipped with an impactor at 45° on the vertical axis to induce normal and tangential accelerations due to the oblique impact [23]. Bland et al. positioned the head in six ways to elicit distinct impact zones, reporting distinct distributions of peak linear accelerations in the different impact regions [23]. Furthermore, the geometrical attributes of the helmets, depending on the region of the head examined, were related to the impact results, following possible discontinuities in the helmet shell and presenting a larger contact area upon impact [23]. They concluded that depending on the impact region, impacts below the test threshold may also cause significant risks [23]. Wu et al. examined the impact energy absorption capacity of construction helmets subjected to repeated impact scenarios using a drop tower by releasing an impactor from different heights, ranging from 0.30 to 2.03 m [24]. In their experiments, they fixated the helmets to an aluminum head form [24]. They confirmed that, while it may not have been observable by visual examination, the helmets underwent significant structural damage when subjected to impacts above their endurance limit [24]. Additionally, they reported that repeated impacts below the endurance limit of the helmet also degraded the shock absorption performance slightly, although the helmets were still deemed safe for use [24]. Wu et al. highlighted the need for a method to assess the safety of a helmet that undergoes light impacts [24]. Recently, Liu et al. introduced a novel origami-based structure that can be very beneficial for developing novel protective equipment, where buckling-regulated topology leverages deployable folding, unit cell arrangement, and pseudo-rigid features to improve overall energy absorption [25]. The above-mentioned exemplary studies represent only a small subset of the current state-of-the-art experimental impact mitigation; however, a gap remains in achieving realistic head surrogacy to accelerate the development of effective sports gear.

Therefore, the primary objective of this study is to develop a head surrogate for realistic tests of protective helmets in sports. The surrogate is produced with soft and hard tissues using additive manufacturing, allowing the bone geometry and properties of the head to be replicated. This paper is structured with four sections (Introduction, Materials and Methods, Experimental Work, and Conclusions) and is focused on the evaluation of the impact behavior of a cycling helmet in comparison with a commercial surrogate (Hybrid III).

## 2. Materials and Methods

### 2.1. Skull Surrogate Design and Manufacturing

**Figure 1:**
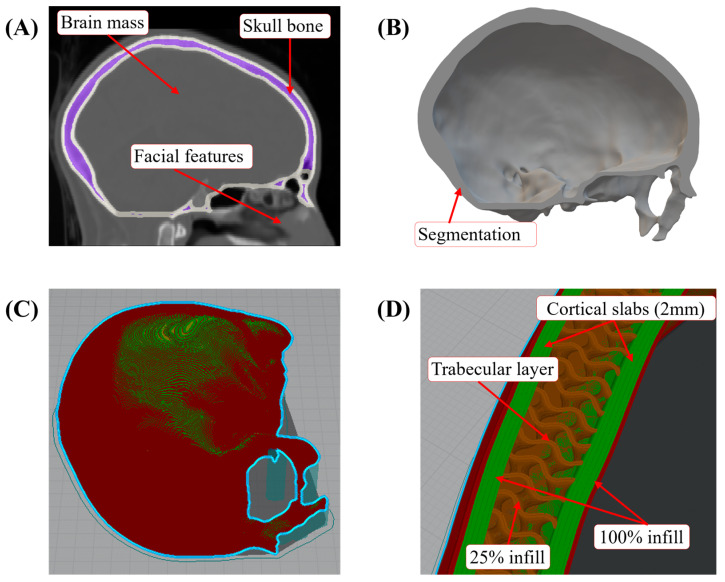
(**A**) Reference CT scan. (**B**) Segmented CAD model with only the skull of the cranial vault. (**C**) Layout of half-skull for 3D printing. (**D**) Geometry and attributes of the trilayer structure.

The skull geometry was developed from computed tomography (CT) scans, where images were segmented using ScanIP V6 software (Simpleware, Exeter, UK) to extract only the bone sections. The segmentation was performed manually by selecting a gray-scale intensity threshold to mark the tissues of interest. The facial bone was omitted from the segmentation since the aim was to replicate the cranial vault, i.e., the region of the skull that serves as a protective and encapsulating element of the brain. Figure 1A shows the scan and the selected features. The threshold segmentation smeared the bony sutures in the CAD model. This simplification is justified since the sutures in the adult skull are completely fused, not allowing relative movement and making them difficult to distinguish in the CT segmentation. In this model, the three-layered structure of the skull bones is maintained with two cortical lamellae enveloping a layer of trabecular bone, including the overall thickness and the relative thickness of each layer according to their anatomical position, as discussed next. The resolved CAD model also neglects some of the internal structures of the skull (e.g., internal bony prominences) and the foramen magnum, given their relevancy to the current study. The final CAD model can be observed in Figure 1B.

The geometrical attributes of the cranial cross-section provide essential contributions to the mechanical behavior of the structure based on the overall wall thickness and infill topology and dimensions. As mentioned above, the total and relative thicknesses of the cranial bone vary throughout to simulate the real structure properly. The latter consists of two superior and inferior plates of dense bone (also called cortical bone), encapsulating a layer of cancellous (trabecular) bone [26]. The personalized skull surrogates emphasize the construction of the superior region (corresponding to the parietal bones) based on the impact loading scenario considered herein, as described in the forthcoming section. Furthermore, the infill percentage is directly related to the density within a specific region to simulate the ratio between the density of trabecular and cortical bones in the skull (~25%). The infill geometry was set as gyroids, given that in several biomedical applications, it is considered to represent the trabecular bone microstructure [27,28]. Figure 1 shows the layout of the hemi-cranium for additive manufacturing (panel C) and the modeling attributes of the three layers of the bone (panel D).

The resolved skull surrogates were fabricated using the material extrusion (MEX) 3D printing process, also referred to as Fused Filament Fabrication or Fused Deposition Modeling [29]. The skulls were 3D-printed using polylactic acid (PLA) filaments with common processing parameters, including a 205 °C extrusion temperature, a 50 °C printing bed temperature, a 60 mm/s printing speed, and a 25% infill. The material selection has grounds in previous studies by the authors [30]. This study also covers the sectioning strategy, accounting for the effects of the orientation of the manufactured parts. The skulls were fabricated in an enclosed 3D printer (Epsilon W50, BCN3D, Barcelona, Spain), given the relatively long printing processes that could lead to noticeable thermal gradients when exposed to ambient airflow [29].

### 2.2. Brain Mass Surrogate

For the brain mass surrogate, a gel-like material was molded directly into the skulls, corresponding to the brain mass and the cranium cavity. A commercial silicone (Ecoflex 0030, SmoothOn, East Texas, PA, USA) was thoroughly mixed in a 1:1 weight ratio before being cast into the 3D-printed skull and left to cure at room temperature for 4 h. The mechanical properties of Ecoflex 0030 were provided by the manufacturer [31].

### 2.3. Demonstrator

**Figure 2:**
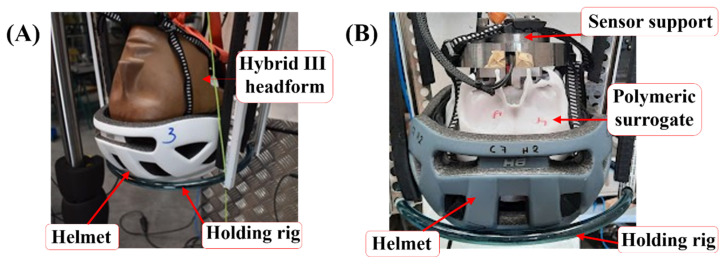
Assembly of demonstrators with cycling helmets of (**A**) Hybrid III and (**B**) surrogate in the drop tower.

A demonstrator was devised to study the feasibility of the standard and 3D-printed head surrogates fitted with commercial cyclist helmets (certified and validated per UNE-EN1078 [19]). The assembled helmet–head, i.e., the helmet with the Hybrid III head-form dummy or the helmet with the 3D-printed silicone-filled skull, were dropped on a blunt steel cylinder with impact velocities ranging between 3.8 m/s and 5.4 m/s, corresponding to fall heights of 0.75 and 1.50 m, respectively. Figure 2A illustrates the positioning of the testing construct assembled on the drop tower guide, where the helmet was buckled to the Hybrid III head form to prevent relative motion between the helmet and head form. Since the 3D-printed skull surrogate lacked a lower facial region, as discussed in Section 2.1, a specifically designed fixture was used to attach the bicycle helmet to the surrogate. A ballast was also attached to the 3D-printed surrogate to normalize the impact energy between the testing constructs. Figure 2B shows the helmet-fitted 3D-printed skull and the ballast in the drop-test configuration. The helmets used for both head forms were of the same mark and model, with the only difference between them being the color, due to commercial availability.

### 2.4. Signal Acquisition and Processing

**Figure 3:**
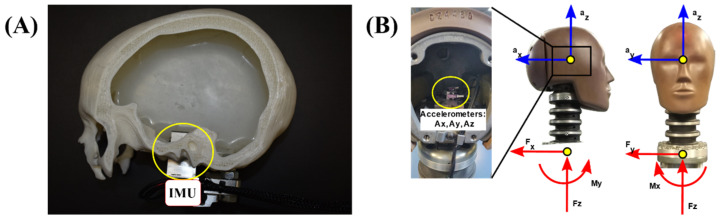
Sensorization of the head forms and location of the accelerometers in (**A**) the 3D-printed head surrogate and (**B**) the Hybrid III.

The newly developed head surrogates were fitted with a 6-axis inertial sensor (IMU, Bay Sensor Tec, GmbH, Eching, Germany), shown in Figure 3A, consisting of a piezoresistive sensor and three aluminum-encapsulated accelerometers. The sensor could record up to 1000 g of linear acceleration at 3 kHz. In the case of the Hybrid III P50 dummy, the integrated sensors were linear accelerometers (TE Model 64C-2000-360 with a range of 2000 g) located at the center of gravity and aligned with the major axes of the head. Figure 3B shows the helmet-saddled Hybrid III head form and the location of the accelerometers. These sensors were connected to the DEWE3-A4 dynamic acquisition system via TRION 1850-MULTI acquisition modules (Dewetron GmbH, Raaba-Grambach, Austria) capable of acquiring data at a sample rate of up to 5 MHz.

## 3. Results and Discussion

### 3.1. Acceleration–Time Histories

**Figure 4:**
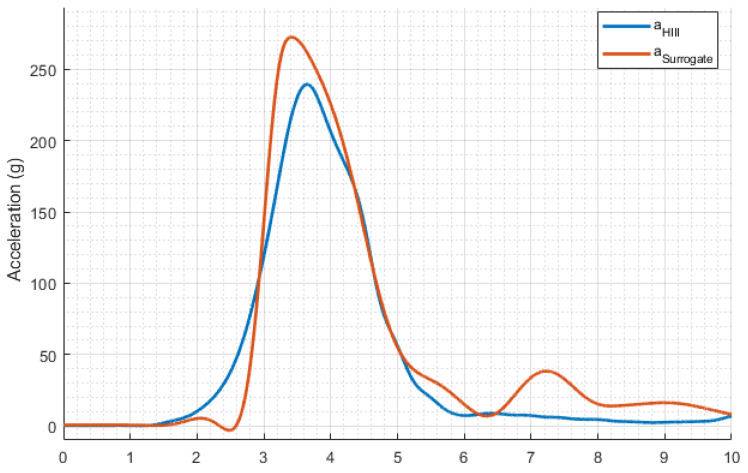
Calibration curves for the Hybrid III head form (blue) and the 3D-printed head surrogate (red).

The calibration procedure demonstrated that the acceleration measurements fell within the specified range, as shown in Figure 4. A low-energy impact was used to confirm the operation of the sensing elements used herein. The Hybrid III head form was dropped from a height of 376 mm, resulting in an impact velocity of ~2.7 m/s. The acceleration resulting from the low-energy impact with the Hybrid III head reached a peak of 239.47 g, indicating that the system was functioning correctly since its acceleration was within the manufacturer’s specifications of 225 g to 275 g. An impact with similar attributes was used to verify the newly fitted, 3D-printed head surrogate, reporting a peak acceleration of 272.70 g. The slight difference between the recorded acceleration of the head forms is attributed to the distinct overall mass and the absence of a skin simulant in the 3D-printed skull. All data channels were filtered using CFC-1000 phase-less filters, according to SAE J211-1 [32], before extracting the peak acceleration.

**Figure 5:**
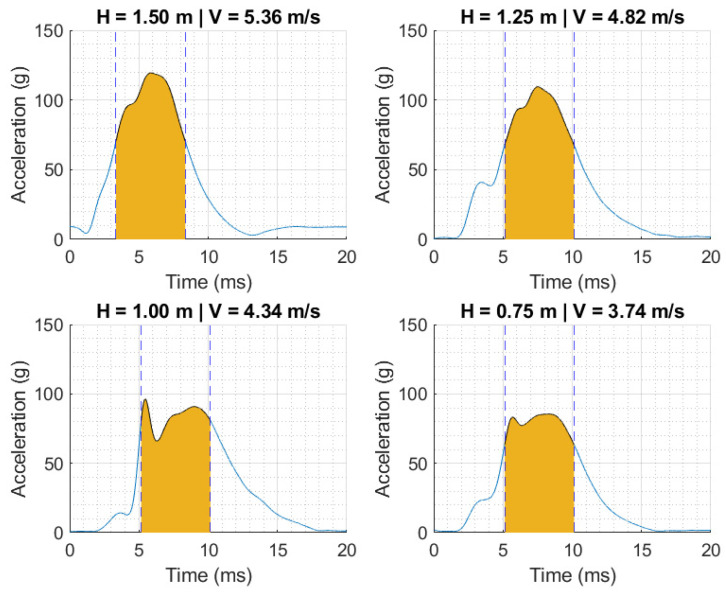
Acceleration–time histories as a function of drop height and velocities using cyclist helmet–Hybrid III testing construct. Shaded region is the time interval that maximizes the value of the HIC.

Figure 5 depicts the acceleration curves resulting from drop impacts conducted on the Hybrid III at four different heights and corresponding velocities. Within each acceleration–time history, a shaded region corresponds to the interval from *t*_1_ to *t*_2_ that maximizes the HIC, which is a widely utilized damage indicator in the analysis and prevention of head injuries [7]. The HIC is calculated based on

(1) $$HIC={\left\{{\left[\frac{1}{\left(t_{2}-t_{1}\right)}\int_{t_{1}}^{t_{2}}a\left(t\right)dt\right]}^{2.5}(t_{2}-t_{1})\right\}}_{max},$$

where *a* is the acceleration as a function of time *t*. The peak acceleration and HIC data extracted from the responses in Figure 5 are summarized and discussed in the next section.

**Figure 6:**
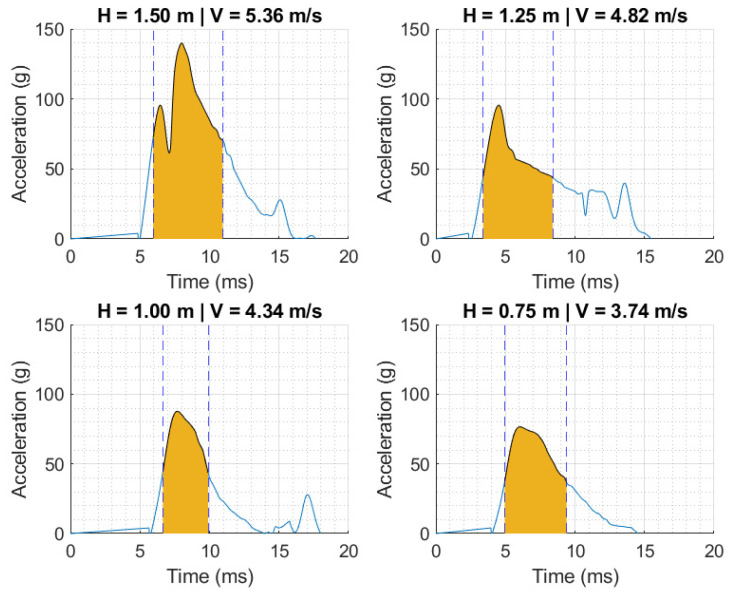
Acceleration–time curves were measured by dropping a 3D-printed, silicone-filled head surrogate fitted with a cyclist helmet at different velocities. Shaded region is the time interval that maximizes the value of the HIC.

Similarly, the measured acceleration–time curves from the helmet/3D-printed skull surrogate assembly are plotted in Figure 6 as a function of drop heights ranging between 0.75 and 1.5 mm. The acceleration–time histories in Figure 6 indicate the manifestation of sharper peaks, pointing to a shorter acceleration pulse than previously observed. Nonetheless, the peak accelerations remained below the acceptable range specified by UNE-EN 1078 [19], i.e., <250 g. The performance metrics from the 3D-printed, silicone-filled skull surrogates are summarized and discussed in the next subsection.

### 3.2. Peak Linear Acceleration and Head Injury Criterion

Table 1 summarizes the kinematic performance metrics for the impact conditions for the helmet–Hybrid III experimental constructs as a function of drop height. Table 2 reports the same metric based on testing the 3D-printed silicone-filled skull under similar conditions. The reported performance metrics include the impact attributes (i.e., height and velocity), peak acceleration (maximum of acceleration–time history), and HIC (calculated using Equation (1)). Irrespective of the head form, the impact velocities ranged between 3.74 m/s and 5.33 m/s, corresponding to peak accelerations of 85 g and 76 g and 119 g and 140 g for the Hybrid III and 3D-printed surrogate, respectively. At intermediate impact velocity, the peak accelerations were 96 g and 88 g, respectively, at a drop velocity of ~4.32 m/s. The calculated HIC values reached maxima of 522 and 478 for the most critical case at the highest fall height, associated with the Hybrid III and 3D-printed head forms, respectively. It is imperative to note that the time intervals for calculating the HIC (Figure 5 and Figure 6) are comparable in duration; hence, the evolution of impact energy is ascribed to changes in the acceleration peak.

The duration relative to the peak pulse is longer for the Hybrid III than for the fabricated simulants. As discussed above, the mechanisms protecting the head from damage incurred after an impact event are related to the value of the acceleration peak and its duration. The HIC, a common indicator to establish damage thresholds for impact scenarios, is based on the integral of the acceleration pulse in the range that maximizes the resulting value (i.e., Equation (1)). Despite the apparent longer pulse duration in some cases, the range for calculating the HIC in all impacts was kept similar to facilitate comparison.

**Figure 7:**
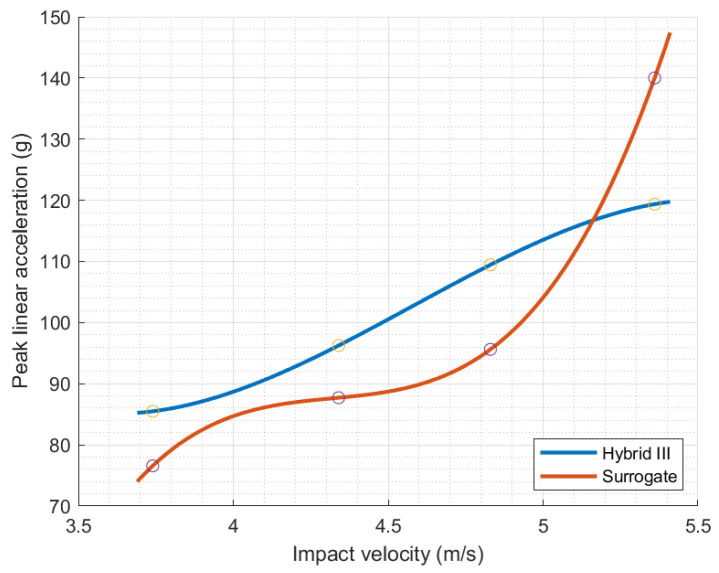
Impact response of the two head forms represented by peak linear acceleration as a function of impact velocity. Datapoints are highlighted in circles.

The peak linear accelerations impacting the head forms considered herein at different velocities are compared in Figure 7, where the acceleration was measured at the center of mass of each head form, as discussed above. The impact response of the surrogate is in reasonable agreement with those reported using the Hybrid III head form while considering the difference in construction and the legacy optimization of the latter. The silicone/3D-printed surrogate also excludes the contribution of important anatomical features, such as the skin and the cervical vertebrae, which are part of the Hybrid III head form. The assembly of the head with the supports of the sensor is also inherently different in the two alternatives. In the case of the Hybrid III, the sensor support is a rigid fixture that is an extension of the titanium case, while in the polymeric surrogate, the sensor support is mounted and attached to the fabricated head. In other words, the distinct construction of the head forms used in this study is generally responsible for the dichotomy in the overall impact response and the extracted performance metrics, as exemplified in Figure 7. The quasi-linear relationship between the peak linear acceleration and impact velocity of the Hybrid III head form is primarily attributed to the linear springs representing the neck. The first generation of the 3D-printed surrogate presented herein for the first time lacks systematic optimization of the overall geometry and specific design details, material properties, manufacturing parameters, and instrumentation placement. Nonetheless, the evolution of the impact response is reasonably similar in both substitutes of the human head, indicating that the first generation of 3D-printed surrogates could be further optimized to readily substitute the state of the art.

## 4. Conclusions

The work presented in this article proposes the development of polymeric head surrogates using additive manufacturing techniques. These surrogates offer a practical and efficient alternative to the standard head forms for analyzing head impact scenarios. The proposed surrogates were developed with the most commonly used material in polymer 3D printing, PLA, for the skull and an easily accessible and characterized commercial silicone for the brain matter. The assemblies made using the head surrogate and the bicycle helmet were subjected to guided free falls in a drop tower from different heights, resulting in different impact energies. A head form from the Hybrid III system was studied to compare the response of the proposed surrogates. These experiments showed the behavior of the head substitutes and enabled the establishment of an equivalence between the mechanical response of the proposed polymeric head surrogates and the commercially employed Hybrid III. This study showcased the feasibility of manufacturing instrumented head surrogates with a reduced cost, enabling the application of additive technologies to reproduce diverse impact cases that are useful in fields related to forensic research. The main conclusions are enumerated below.

1. The skull surrogates were developed using AM techniques, highlighting the viability of these methodologies for readily fabricating experimental constructs required for assessing sports protection equipment. AM is suitable for overcoming the challenges stemming from complex geometries and material properties.
2. The proposed 3D-printed surrogate is agnostic to the specific anatomical features and can be easily adapted to analyze the performance of other sports gear while accounting for variability caused by gender, age, and other physiological parameters.
3. The performance of the developed surrogates was compared to the standardized Hybrid II head form, showing reasonable agreement in the overall acceleration–time histories and the extracted impact efficacy metrics (i.e., peak linear acceleration and HIC). These results analogize and validate the two substitutes of the human head considered herein.

Future research will focus on further improving the accuracy of the 3D-printed surrogate by including important anatomical features, making the fabricated surrogate faithful to real-life experimental conditions.

## Figures and Tables

**Table 1:** Kinematic parameters of the impacts measured in the Hybrid III.

Drop Height	Impact Velocity	Peak Linear Acceleration	HIC
1.50 m	5.36 m/s	119 g	522
1.25 m	4.82 m/s	109 g	425
1.00 m	4.34 m/s	96 g	320
0.75 m	3.74 m/s	85 g	286

**Table 2:** Kinematic parameters of the impacts measured in the head surrogate.

Drop Height	Impact Velocity	Peak Linear Acceleration	HIC
1.50 m	5.33 m/s	140 g	478
1.25 m	4.82 m/s	95 g	151
1.00 m	4.31 m/s	88 g	144
0.75 m	3.74 m/s	76 g	133

## Data Availability

The original contributions presented in the study are included in the article, further inquiries can be directed to the corresponding author.
